# Open‐Source CFD Elucidating Mechanism of 3D Pillar Electrode in Improving All‐Solid‐State Battery Performance

**DOI:** 10.1002/advs.202105454

**Published:** 2022-02-08

**Authors:** Weizhuo Li, Zhiming Bao, Qing Du, Yifan Xu, Kui Jiao

**Affiliations:** ^1^ State Key Laboratory of Engines Tianjin University 135 Yaguan Rd Tianjin 300350 China

**Keywords:** 3D pillar electrodes, all‐solid‐state batteries, contact area, electrochemical model, OpenFOAM

## Abstract

All‐solid‐state batteries (ASSBs) have become an important technology because of their high performance and low‐risk operation. However, the high interface resistance and low ionic conductivity of ASSBs hinder their application. In this study, a self‐developed electrochemical model based on an open‐source computational fluid dynamics platform is presented. The effect of contact area reduction at the electrode/solid‐state electrolyte interface is investigated. Then, a new conceptual 3D structure is introduced to circumvent the existing barriers. The results demonstrate that the discharge time is shortened by over 20% when the area contact ratio reduces from 1.0 to 0.8 at 1 C‐rate, owing to the increased overpotential. By adopting the new 3D pillar design, the energy density of ASSBs can be improved. However, it is only when a 3D current collector is contained in the cathode that the battery energy/power density, capacity, and material utilization can be greatly enhanced without being limited by pillar height issues. Therefore, this work provides important insight into the enhanced performance of 3D structures.

## Graphical abstract

This work presents a self‐developed electrochemical model for all‐solid‐state batteries (ASSBs) based on an open‐source computational fluid dynamics platform, and introduces a new conceptual 3D pillar structure to enhance ASSB performance. The new design with a 3D current collector improves cell capacity significantly with increasing pillar height without loss of power density, making this a promising structure for practical applications.

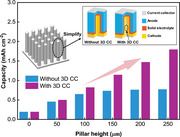

## 1 Introduction

Increasing global energy shortages and environmental pollution have promoted the utilization of renewable energy and the electrification of transportation.^[^ ^1^ ^]^ As a result, the lithium‐ion battery (LIB) market has seen rapid growth and development. However, mainstream LIBs with organic liquid electrolytes are widely believed to be approaching their theoretical limits of energy density.^[^ ^2^ ^]^ Meanwhile, incidents resulting from the flammability and volatility of organic liquid electrolytes have occurred frequently in recent years.^[^ ^3^, ^4^ ^]^ By replacing the liquid electrolytes with solid‐state electrolytes (SSEs), the recently developed all‐solid‐state batteries (ASSBs) are regarded as a promising candidate for the next‐generation LIB technology.^[^ ^5^ ^]^ The SSEs can be combined with lithium metal anodes and high‐voltage cathode materials to significantly improve the battery energy/power density.^[^ ^6^, ^7^, ^8^ ^]^ However, so far, the low ionic conductivity in the SSEs and the high interface resistance at the SSE/electrode interfaces remain barriers to large‐scale applications.^[^ ^9^, ^10^ ^]^

Electrochemical modeling, which can describe the electrochemical distribution characteristics inside a full cell, has been widely used in the performance prediction and structure optimization of LIBs with porous electrodes and liquid electrolytes.^[^ ^11^, ^12^, ^13^, ^14^, ^15^ ^]^ Recently, studies on the mathematical modeling of ASSBs have been reported.^[^ ^16^, ^17^, ^18^, ^19^, ^20^, ^21^, ^22^, ^23^ ^]^ However, describing the cell potential by Poisson's equation instead of directly modeling charge transport is not conducive to 3D modeling.^[^ ^16^ ^]^ SSEs can be divided into binary‐ion conductors and single‐ion conductors based on whether both lithium cations and anions can move or not.^[^ ^17^ ^]^ The battery with a single‐ion conducting electrolyte has an advantage over that with a traditional binary electrolyte, especially at high discharge rates, due to the large concentration polarization in the latter.^[^ ^18^ ^]^ When modeling lithium‐ion transport in a single‐ion conducting electrolyte, a uniform distribution of lithium‐ion is usually assumed.^[^ ^19^, ^20^ ^]^ In contrast, Danilov et al.^[^ ^21^ ^]^ presented a new single‐ion electrolyte model in which lithium may be present in two types of states (i.e., oxygen‐bonded lithium and mobile lithium‐ion), and the ionic conduction process is dominated by ions. The transfer process between the two states of lithium was described by an ionization reaction. Additionally, the diffusion coefficient in electrodes is also an important property that directly determines the model accuracy; it is concentration dependent but often set as a constant in the model development, hindering the further improvement of model accuracy, especially under high current rates.^[^ ^22^, ^23^ ^]^ In short, there is still plenty of scope for developing a more widely applicable ASSB model with better accuracy.

**Figure 1:**
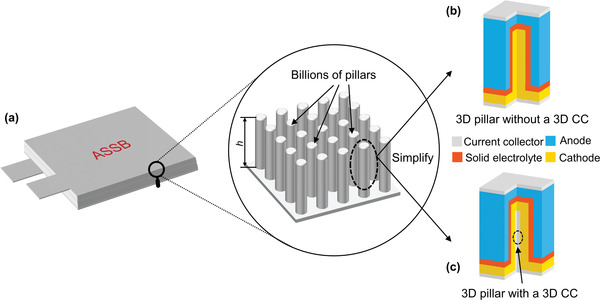
Schematic diagram of the new conceptual 3D all‐solid‐state battery. a) A new conceptual 3D all‐solid‐state battery and detail view; b) A representative unit without a 3D current collector; c) A representative unit with a 3D current collector.

The imperfect physical contact of the solid–solid interface between the SSE and the electrodes is one of the major issues for ASSBs. The battery capacity drop in ASSBs is correlated with the reduced contact area.^[^ ^24^ ^]^ In composite electrodes, the void spaces between the two solid phases impede the transportation of lithium‐ion and can result in an inhomogeneous distribution of the ions. However, the void spaces can also serve as buffer spaces that accommodate the expansion of active materials and reduce stress formation.^[^ ^25^, ^26^ ^]^ Due to the low ionic conductivity of SSEs, ASSBs are generally thin‐film structures that shorten the lithium‐ion transport distance, thereby offering a low energy/power density in a confined area. In this study, we introduce 3D structures to simultaneously increase the contact area and volume of active materials. The 3D structures were initially used to address the power density requirement of microelectronic devices, where the active material was stored in the vertical direction to increase the reaction surface area.^[^ ^27^ ^]^ The reported 3D structures in the literature include 3D interdigitated^[^ ^28^, ^29^ ^]^, 3D trench^[^ ^30^ ^]^, and 3D concentric^[^ ^31^, ^32^ ^]^. The 3D interdigitated structure consists of alternating arrays of cylindrical anodes and cathodes, which are attached to two separate bases and filled with an SSE. The charge transport in the 3D interdigitated structure is inhomogeneous, which may result in the low utilization of active materials and the risk of local over‐charge/discharge. Among the aforementioned structures, heat generation is most prominent in the 3D trench, and the performances of all the three structures are limited by their pillar heights. In contrast, Clancy et al.^[^ ^33^ ^]^ designed two 3D structures and compared their performance with a planar thin‐film structure. However, the 3D structures had a long distance between opposite electrodes, which amplifies the disadvantage of the low ionic conductivity of SSEs. The battery manufacturer LionVolt recently demonstrated a new conceptual 3D ASSB with a capacity of 12.5 Ah for use in electric vehicles, which was made by covering billions of micropillars with thin layers of functional material.^[^ ^34^ ^]^ Each pillar can be regarded as a miniature battery with a short distance for lithium‐ion transport. However, the internal physical field distribution for this analogous new structure (**Figure** **1**) needs to be further investigated if performance improvements are to be made.

In this paper, we developed a 3D layered electrochemical model for ASSBs by coupling charge transfer kinetics at the SSE/electrode interfaces, lithium‐ion transport in the SSE and positive electrode based on an open‐source computational fluid dynamics (CFD) platform, OpenFOAM. A concentration‐dependent diffusion coefficient was used for the cathode to improve the model accuracy. Using our advanced model, we systematically studied the effect of contact area reduction in terms of discharge curves, charge‐transfer overpotential, and diffusion overpotential. A representative unit for the new conceptual 3D ASSB was then chosen to compare the design with or without a 3D current collector (CC) (Figures 1b,c). Our results show that the new design with a 3D CC can significantly enhance ASSB performance with respect to energy/power density, capacity, and material utilization, which makes it a promising structure for practical applications.

## 2 Results and Discussion

### 2.1 Effect of Contact Area Reduction

**Figure 2:**
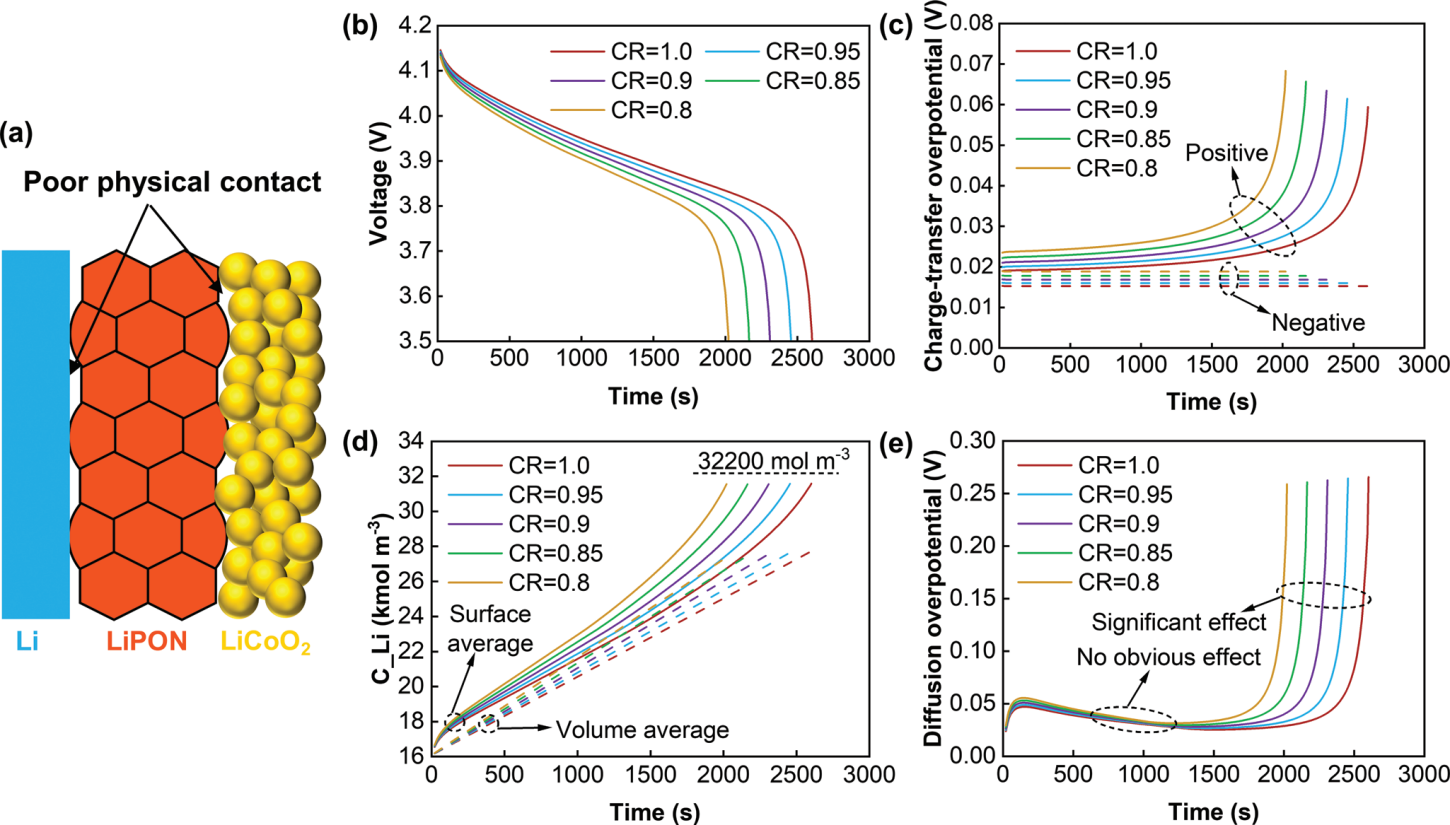
Effect of contact area ratio on all‐solid‐state battery discharge behaviors at 1 C. a) Bad interface contact between electrodes and solid‐state electrolyte; b) Discharge curves; c) Charge‐transfer overpotential; d) The average lithium concentration at the positive interface and electrode; e) Diffusion overpotential.

Unlike liquid electrolytes, which readily wet electrode surfaces, SSEs exhibit high electrical resistance at the interface with electrodes due to poor physical contact (**Figure** **2**a). With a reduced contact area, the entire discharge voltage curve is lowered and the discharge time is shortened (Figure 2b). For an area contact ratio (CR) of 0.8, the discharge time with a cut‐off voltage of 3.5 V is shortened by ≈22.1% compared with the perfect contact. The drop in voltage can be attributed to the increase in the charge‐transfer overpotential between the SSE and electrode, whereas the reduction in discharge time is attributed to the fast accumulation of intercalated lithium at the positive electrode interface.

The charge‐transfer overpotential for the anode remains nearly constant during discharging, while that for the cathode exponentially increases with the deepening of discharge due to the increasing concentration polarization (Figure 2c). In addition, the cathode has a larger contribution to the total charge‐transfer overpotential than the anode. As the contact area decreases, the entire interface overpotential gradually increases, because a smaller reaction area requires more driving force under the same current flux.

In addition, the loss of contact area can increase the concentration of intercalated lithium at the interface, because the amount of lithium produced is the same at a reduced surface area (Figure 2d). The average lithium concentration at the SSE/positive electrode interface gradually increases from 16 100 to 32 200 mol m^–3^ during the discharge process. It is noteworthy that the accumulation rate is accelerated in the second half of the discharge because of the reduced diffusion coefficient, while the volume average concentration linearly increases. In addition, the accumulation rate of the average lithium concentration increases with the loss of contact area. Subsequently, a scalar called diffusion overpotential is defined to quantitatively describe this effect (Figure 2e). It is seen, notably in the second half of the discharge process, that the smaller the contact area ratio, the faster the overpotential increase rate. Comparing Figure 2c,e, the diffusion overpotential has a matched value with the charge‐transfer overpotential in the first half of the discharge but dominates at the end of discharge.

### 2.2 3D Pillar Structure

Increasing the electrode thickness is a straightforward method to enhance the performance of thin‐film ASSBs. However, this also increases the lithium‐ion transport path and diffusion loss, causing a reduction in the power density (Note S1, Supporting Information). Therefore, a new conceptual 3D pillar electrode has been designed to increase the contact area and the quantity of active materials. The effects of variation in pillar height on the performance of the battery with two new 3D designs (without a 3D CC and with a 3D CC) were studied. The thicknesses of the electrodes, SSE, and CC layers for the two new designs were identical.

**Figure 3:**
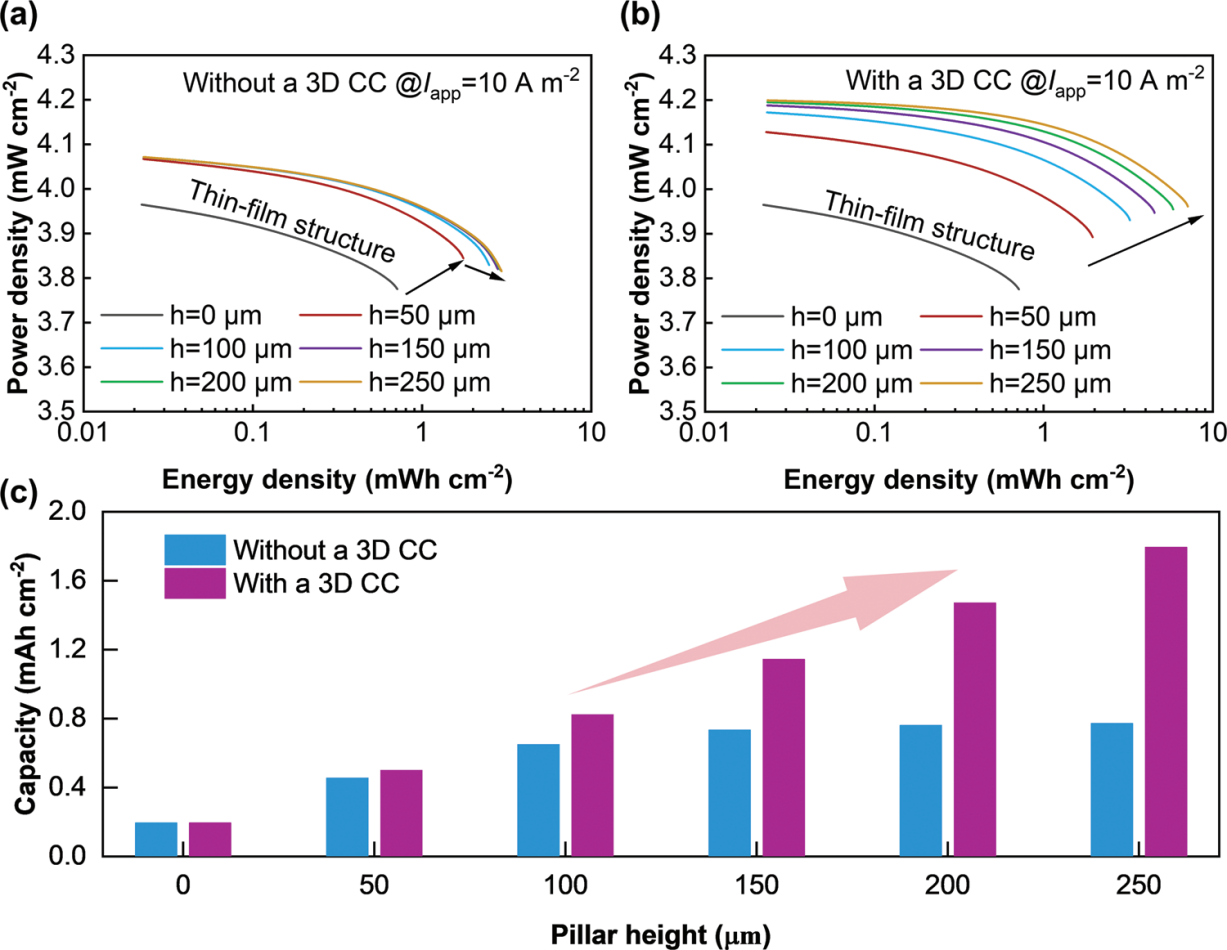
Performance of the new 3D pillar structures. a) Ragone plot of the new 3D design without a 3D current collector; b) Ragone plot of the new 3D design with a 3D current collector; c) Comparison of discharge capacity for two new 3D electrode designs.

**Figure** **3**a,b shows the Ragone plot of two new designs for various pillar heights compared to a basic thin‐film structure. The power density is significantly lowered with an increase in the energy density due to the decay of battery voltage in the discharge process. For the new design without a 3D CC (Figure 3a), both the energy density and power density initially increase with pillar height compared to the thin‐film structure. However, the energy density growth rate gradually slows to near zero, and the power density decreases slightly at pillar heights above 100 µm. For example, compared with the thin‐film structure, the energy densities for pillar heights of 50, 100, 150, and 200 µm are enhanced by 2.456, 3.490, 3.928, and 4.074 times, respectively. The corresponding power density at the end of discharge improves from 3.775 to 3.844 mW cm^–2^ (at 50 µm) before decreasing back down to 3.817 mW cm^–2^ (at 250 µm). This behavior can be explained as follows. As the pillar height increases, more active material can be stored with a larger electrochemical reaction surface area, which results in a higher energy density. However, the transport pathway of current to the base layer also becomes longer with increasing pillar height, which, together with the low conductivity of LiCoO_2_, causes a larger ohmic loss in the pillar electrode. This design flaw can be overcome using a 3D CC on the positive side (Figure 3b). Both the energy density and power density are continuously improved by increasing the pillar height from 50 to 250 µm. The energy density is increased up to a maximum of 8.165 times that of the thin‐film structure, and the relevant power density increases from 3.775 up to 3.955 mW cm^–2^ (at 250 µm). Adopting the new design with a 3D CC, the battery performance can be significantly enhanced by increasing the pillar height without being constrained by the large internal resistance in the height direction.

The capacities of the two new designs are similar at low pillar heights (e.g., 50 µm) (Figure 3c). However, when the pillar height is increased above 100 µm, the corresponding capacity of the design without a 3D CC does not improve significantly. Conversely, the capacity of the new design with a 3D CC increases linearly with pillar height, with a 9.23‐times increase at a pillar height of 250 µm. In brief, the new design without a 3D CC is recommended for smaller capacity and power requirements because of its comparatively simple structure, while the new design with a 3D CC can be applied to meet higher capacity and power requirements.

**Figure 4:**
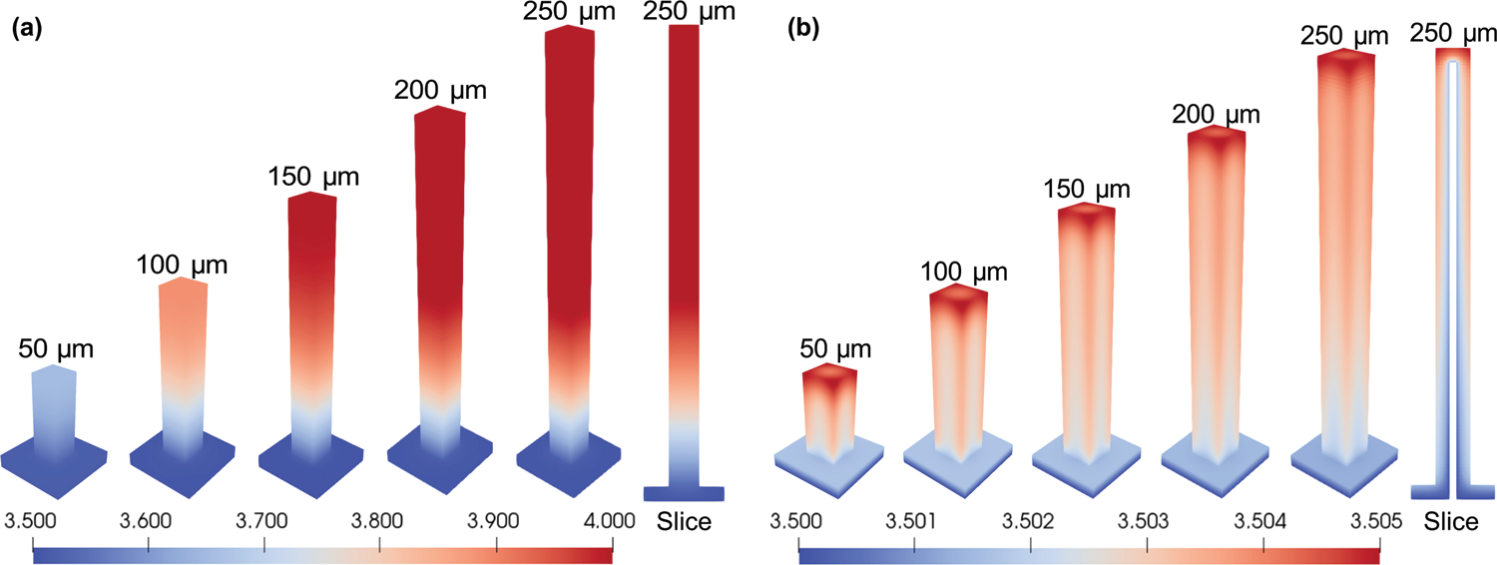
Potential distribution at the end of discharge: a) without a 3D current collector, b) with a 3D current collector.

To investigate the reason for the different effects of pillar height on the above two new designs, we measured the local distributions of potential in the positive electrode for the new design with and without a 3D CC (**Figure** **4**). The potential in the new design without a 3D CC decreases from the top of the pillar to the base layer, which implies that the current transport pathway in the positive electrode is along the pillar height direction (Figure 4a). Owing to the low conductivity of LiCoO_2_, the maximum potential difference in the positive electrode significantly increases with increasing pillar heights, with a value as high as 0.5 V when the pillar height is 200 µm. In contrast, the maximum potential difference in the positive electrode of the new design with a 3D CC is always small across all the pillar heights studied (Figure 4b). There is a potential gradient along the pillar height direction, but the main potential gradient is along the thickness of the electrode layer. As a result, the current can pass through the low conductivity positive electrode at the shortest distance and travel through the high conductivity 3D CC along the height direction, minimizing ohmic loss in the pillar electrode.

**Figure 5:**
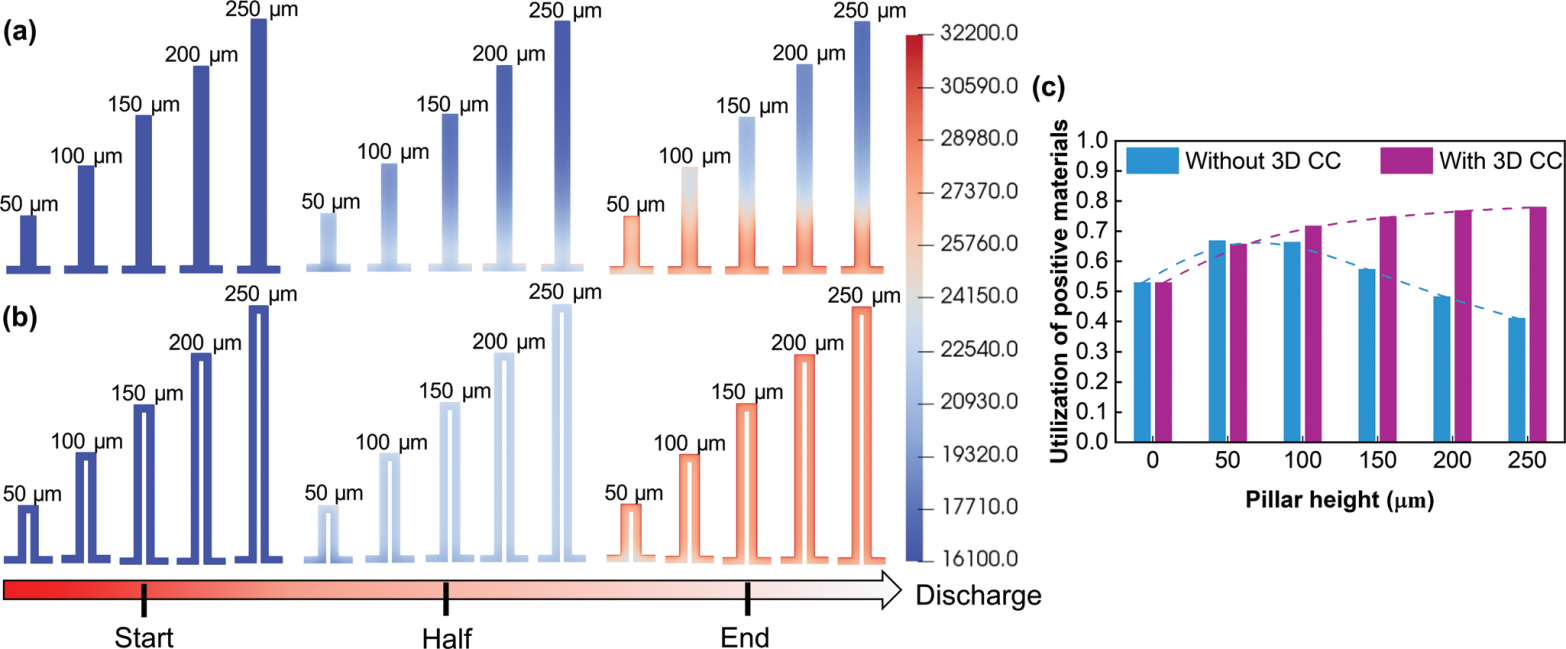
Transport properties of positive electrode: a) depth of lithiation development for the new 3D structure without a 3D current collector, b) depth of lithiation development for the new 3D structure with a 3D current collector, c) utilization of positive active materials for two new 3D designs.

The various impacts of the above two new designs can also be understood by the concentration of intercalated lithium‐ion in the positive electrode (**Figure** **5**a,b). For the new design without a 3D CC (Figure 5a), the lithiation process for a pillar height of 50 µm starts synchronizing near the interface and then diffuses inward, while the lithiation of greater pillar heights mainly take place near the base layer. For a pillar height of 200 µm, approximately half of the pillar electrode is not well utilized at the end of discharge because of the dominant ohmic loss. By introducing a 3D CC (Figure 5b), the lithiation processes are almost identical when the pillar height is changed from 50 to 250 µm, and have the same trend as the new design without a 3D CC with a height of 50 µm. Additionally, the lithiation depth and overall uniformity improve with increasing pillar height because of the larger reaction interface area. Hence, the material utilization of the new design with a 3D CC is enhanced (Figure 5c) from 0.53 to 0.78 with increasing pillar height from 0 to 250 µm. For the new design without a 3D CC, by changing the pillar height from 0 to 50 µm, the material utilization is raised from 0.53 to 0.67. However, further increasing the pillar height from 50 to 250 µm decreases the material utilization from 0.53 to 0.41. This is due to the balance of the positive effect of increasing the reaction area and the more dominant negative effect of more underutilized active material.

## 3 Conclusions

In summary, to systematically understand the effect of contact area reduction and the contribution of new 3D structures to ASSBs, we independently developed a 3D layered electrochemical model based on an open‐source CFD platform, OpenFOAM. With the deepening of discharge, the diffusion overpotential occupies the dominant position in the total losses from a value equivalent to the charge‐transfer overpotential. The poor contact between the electrode and SSE causes a large charge‐transfer overpotential and diffusion overpotential, which leads to a rapid drop in the discharge voltage. Adopting the new 3D structure can increase the reaction area and boost the battery performance. We find that the maximum benefit of the design without a 3D CC is limited due to the large ohmic loss and low material utilization at greater pillar heights. Conversely, the introduction of a 3D CC can maintain the ohmic loss in the cathode at a constant small value while increasing material utilization at greater pillar heights. These results may advance the understanding of 3D structures and suggest that the 3D pillar design with a 3D CC is a promising structure for enhancing the performance of ASSBs.

## 4 Experimental Section

### Governing Equations

The electrochemical model for ASSBs was developed mainly based on mass conservation equation, charge conservation equation, and interface electrochemical reaction equation, as follows:

(1) $$\frac{\partial c_{\mathrm{Li},\mathrm{pe}}}{\partial t}=\nabla \cdot \left(D_{\mathrm{Li},\mathrm{pe}}\nabla c_{\mathrm{Li},\mathrm{pe}}\right)+S_{\mathrm{Li},\mathrm{pe}}$$

(2) $$\frac{\partial c_{Li^{+}}}{\partial t}=\nabla \cdot \left(D_{Li^{+}}\nabla c_{Li^{+}}\right)+S_{Li^{+}}+S_{Li^{+}}^{\mathrm{ionize}}$$

(3) $$\nabla \cdot \left(\sigma_{e}\nabla \phi_{e}\right)+S_{e}=0$$

(4) $$\nabla \cdot \left(\sigma_{\mathrm{ion}}\nabla \phi_{\mathrm{ion}}-\frac{2RT\sigma_{\mathrm{ion}}}{F}\left(1-t_{+}\right)\nabla \left(\ln\left(c_{Li^{+}}\right)\right)\right)+S_{\mathrm{ion}}=0$$

(5) $$j_{\mathrm{loc},i}=\gamma j_{0,i}\left(\exp\left(\frac{\alpha_{a}F\eta_{i}}{RT}\right)-\exp\left(-\frac{\alpha_{c}F\eta_{i}}{RT}\right)\right)$$

where *c*_Li,pe_ (mol m^–3^), *D*_Li,pe_ (m^2^ s^–1^), and *S*_Li,pe_ (mol m^–3^ s^–1^) are the intercalated lithium‐ion molar concentration, diffusion coefficient, and intercalation rate in the positive electrode, respectively;$c_{Li^{+}}$(mol m^–3^),$D_{Li^{+}}$(m^2^ s^–1^),$S_{Li^{+}}$(mol m^–3^ s^–1^), and$S_{Li^{+}}^{\mathrm{ionize}}$(mol m^–3^ s^–1^) are the mobile lithium‐ion molar concentration, diffusion coefficient, generation rate combining reaction generation and ion migration, and generation rate caused by ionization in the SSE, respectively;*φ*_e_(V), *σ*_e_ (S m^–1^), and *S*_e_ (A m^–3^) are the electron potential, conductivity, and generation rate, respectively;*φ*_ion_(V), *σ*_ion_ (S m^–1^), and *S*_ion_ (A m^–3^) are the ion potential, conductivity, and generation rate, respectively; *t*_+_ is the transfer coefficient; *j*_loc,i_ (A m^–2^), *j*_0,i_ (A m^–2^), *γ*, and *η*_i_ (V) are the exchange current density, reference exchange current density, contact area ratio, and overpotential, respectively. The more detailed model development can be seen in Note S2 (Supporting Information).

### Model Framework and Experimental Verification

**Figure 6:**
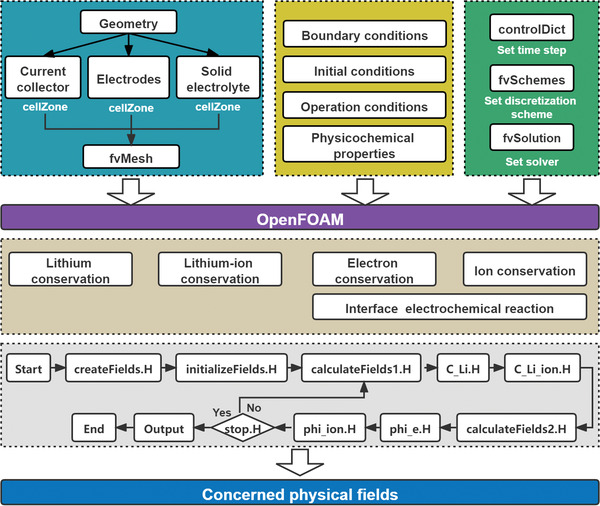
Framework of the developed all‐solid‐state battery model based on OpenFOAM.

**Figure** **6** shows the framework of the self‐developed ASSB model based on open‐source CFD platform, OpenFOAM 7. This software has incomparable advantages of free use, code visibility, flexibility, and high parallel efficiency. Both self‐developed solver and self‐customized case were prepared to simulate the transport and reaction processes in the whole cell, including current collectors, electrodes, and SSE. A case generally incorporated three sub‐parts for primarily storing mesh file, boundary conditions, solver‐settings, etc., which can be changed by a user on demand. The solver was the core of model development that consists of a set of algorithms, describing the transport of lithium‐ion in the electrode and SSE, charge conservation, and interface electrochemical reaction. This solver comprised of a main file and nine inclusion files for clarity of model architecture. The functions of nine inclusion files were scalar/vector fields creation, physical fields initialization, lithium‐ion species fields calculation, intercalated lithium‐ion conservation equation, lithium‐ion conservation equation, potential fields calculation, electron conservation equation, ion conservation equation, and termination condition in sequence, respectively. The solver first read the files in the case and ran the case according to user settings, and finally provided the output according to the concerned fields. The model verification is provided in Note S3 (Supporting Information).

## Conflict of Interest

The authors declare no conflict of interest.

## Author Contributions

K. J. supervised the whole project. K. J., Q. D., and W. L. conceived the idea. W. L. developed the model and carried out the simulation. Z. B. and Y. X. helped solve some modeling problems. W. L. wrote the original manuscript. K. J. provided critical feedback and advice. All authors contributed to the writing and commented on the manuscript.

## Supporting information

Supporting Information

## Data Availability

The data that support the findings of this study are available from the corresponding author upon reasonable request.
