# Supplementary material for: Open‐Source CFD Elucidating Mechanism of 3D Pillar Electrode in Improving All‐Solid‐State Battery Performance
Source: Adv Sci (Weinh). 2022 Feb 8;9(13):2105454. doi: 10.1002/advs.202105454 (PMC9069203; doi:10.1002/advs.202105454)
Supplement: Supplementary file 1 — Supporting Information [file ADVS-9-2105454-s001.pdf]

ADVANCED SCIENCE
Open Access

Supporting Information

for *Adv. Sci.*, DOI 10.1002/advs.202105454

Open-Source CFD Elucidating Mechanism of 3D Pillar Electrode in Improving All-Solid-State Battery Performance

Weizhuo Li, Zhiming Bao, Qing Du, Yifan Xu and Kui Jiao**

ADVANCED SCIENCE
Open Access

Supporting Information

for *Adv. Sci.*, DOI: 10.1002/advs.202105454

Open-source CFD elucidating mechanism of 3D pillar electrode in improving all-solid-state battery performance

Weizhuo Li, Zhiming Bao, Qing Du, Yifan Xu, and Kui Jiao**

Supporting Information

Open-source CFD elucidating mechanism of 3D pillar electrode in improving all-solid-state battery performance

Weizhuo Li, Zhiming Bao, Qing Du*, Yifan Xu, and Kui Jiao*

State Key Laboratory of Engines, Tianjin University, 135 Yaguan Rd, Tianjin 300350, China

*Corresponding authors, e-mail: duqing@tju.edu.cn; kjiao@tju.edu.cn

Note S1 Effect of positive electrode thickness

Figure S1 shows the effect of positive electrode thickness on the power/energy density of the thin-film structure. While the energy density exhibits a slight increase with the positive electrode thickness, the power density descends owing to the sluggish lithium-ion diffusion at cathode. When the electrode thickness varies from 8.08 to 20.2 μm, the energy density increases from 0.732 to 1.016 mWh cm^{-2}, while on the other hand, the power density decreases from 3.765 to 3.730 mW cm^{-2}. Consequently, it is not applicable to improve cell performance by directly increasing electrode thickness.

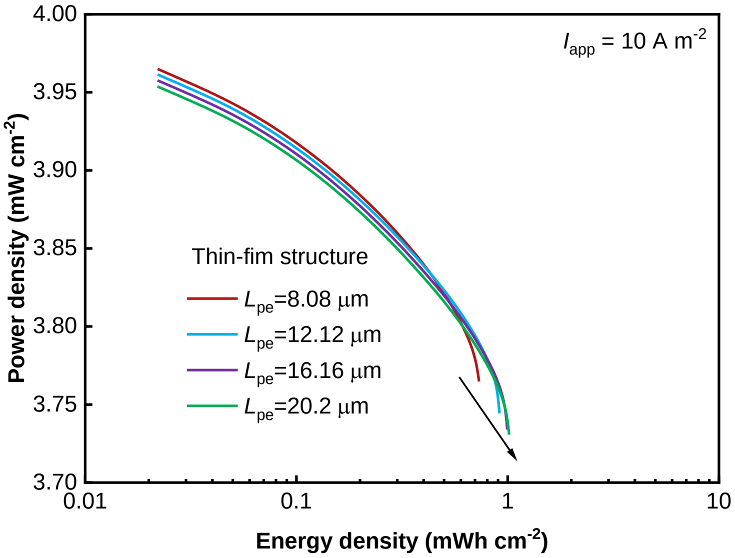

Figure S1. Effect of positive electrode thickness for the thin-film structure.

Note S2 Model development

In this model, the computational domain includes the negative current collector (Cu), negative electrode (Li metal), solid-state electrolyte (SSE) (LiPON), positive electrode ($LiCoO_2$), and positive current collector (Al) (Figure S2), among which the current collectors are necessary to be considered to simulate the non-uniform current distribution in the whole cell as well as the 3D electrode. During the discharge process of an ASSB, the lithium-ion extracted from the lithium metal anode at the negative electrode/SSE interface enters SSE, and is then inserted into the $LiCoO_2$ cathode through the positive electrode/SSE interface. The electrochemical reactions at the electrode/SSE interfaces can be written as follows:

Negative

$$\mathrm{Li} \underset{\text{Charge}}{\overset{\text{Discharge}}{\rightleftarrows}} \mathrm{Li}^{+} + \mathrm{e}^{-} \qquad \text{(Equation S1A)}$$

Positive

$$\mathrm{LiCoO_2} \underset{\text{Discharge}}{\overset{\text{Charge}}{\rightleftarrows}} \mathrm{Li}_{1-x}\mathrm{CoO_2} + x\mathrm{Li}^{+} + \mathrm{e}^{-} \; (0.5 \le x \le 1.0) \qquad \text{(Equation S1B)}$$

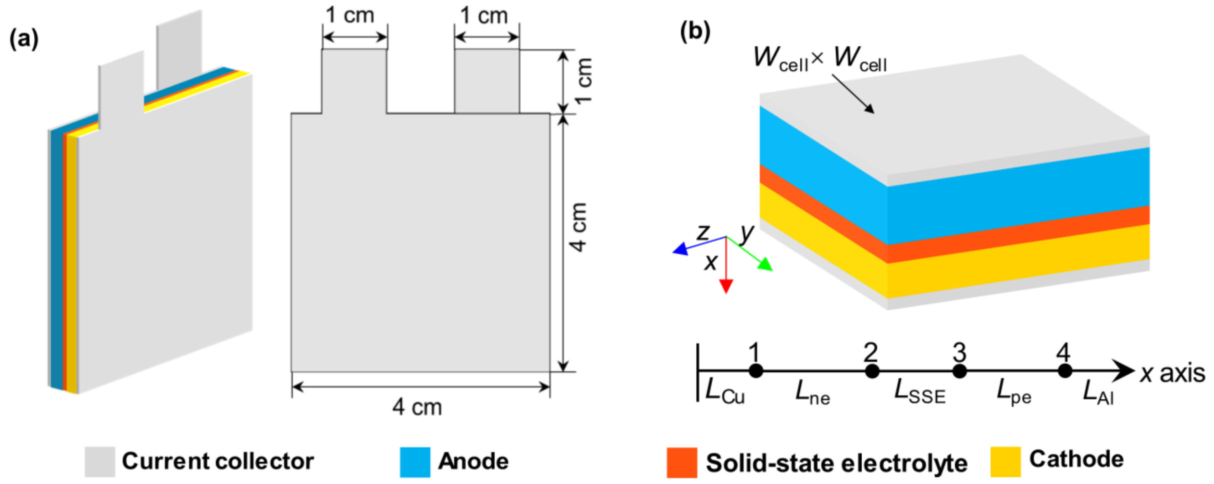

Figure S2 Schematic diagram of thin-film all-solid-state battery. (a) Whole battery; (b) Partial battery.

Herein, a three-dimensional layered electrochemical model is developed to simulate the discharge characteristics of ASSBs.

Assumptions

(1) The electrodes are assumed to be non-porous, and the electrochemical reactions only occur at the interfaces between SSE and electrodes.

(2) A uniform distribution of lithium-ion in the lithium metal anode is assumed, thus the related lithium-ion diffusion process at anode is not considered.

(3) Binary electrolyte and electroneutrality are assumed for SSE.

(4) All side reactions are neglected, and all domains are considered isothermal.

Governing equations

Intercalation lithium conservation (solved in positive electrode)

$$\frac{\partial c_{\mathrm{Li,pe}}}{\partial t} = \nabla \cdot \left(D_{\mathrm{Li,pe}} \nabla c_{\mathrm{Li,pe}} \right) + S_{\mathrm{Li,pe}} \qquad \text{(Equation S2)}$$

where $c_{\mathrm{Li,pe}}$ (mol m^{-3}) is the molar concentration of intercalation lithium, $D_{\mathrm{Li,pe}}$ (m^2 s^{-1}) the diffusion coefficient of intercalation lithium, $S_{\mathrm{Li,pe}}$ (mol m^{-3} s^{-1}) the generation rate of intercalation lithium at the positive electrode/SSE interface.

Lithium-ion conservation (solved in SSE)[1,2]

$$\frac{\partial c_{\mathrm{Li^+}}}{\partial t} = \nabla \cdot \left(\frac{2D_+ D_-}{D_+ + D_-} \nabla c_{\mathrm{Li^+}} \right) + S_{\mathrm{Li^+}} + S_{\mathrm{Li^+}}^{\mathrm{ionize}} \qquad \text{(Equation S3)}$$

where $c_{\mathrm{Li^+}}$ (mol m^{-3}) is the lithium-ion molar concentration, D_+, D_- (m^2 s^{-1}) the diffusion coefficient of cation and anion in SSE, respectively, $S_{\mathrm{Li^+}}$ (mol m^{-3} s^{-1}) the source term of lithium-ion at the electrode/SSE interfaces considering reaction generation and ion migration, $S_{\mathrm{Li^+}}^{\mathrm{ionize}}$ (mol m^{-3} s^{-1}) the ionization generation rate of lithium-ion in SSE.

Electronic charge (solved in negative and positive electrodes and current collectors)

$$\nabla \cdot \left(\sigma_{\mathrm{e}} \nabla \phi_{\mathrm{e}} \right) + S_{\mathrm{e}} = 0 \qquad \text{(Equation S4)}$$

where ϕ_e (V) is the electronic potential, σ_e (S m^{-1}) the electronic conductivity, S_e (A m^{-3}) the electron generation rate at the electrode/SSE interfaces.

Ionic charge (solved in SSE)

$$\nabla \cdot \left(\sigma_{\text{ion}} \nabla \phi_{\text{ion}} - \frac{2RT\sigma_{\text{ion}}}{F} \left(1 - \frac{D_+}{D_+ + D_-} \right) \nabla \left(\ln \left(c_{\text{Li}^+} \right) \right) \right) + S_{\text{ion}} = 0 \qquad \text{(Equation S5)}$$

where ϕ_{ion} (V) is the ionic potential, σ_{ion} (S m^{-1}) the ionic conductivity, S_{ion} (A m^{-3}) the ion generation rate at the electrode/SSE interfaces.

The source terms of mentioned governing equations are listed in Table S1.

Electrochemical reaction kinetics

The electrochemical reaction rates (A m^{-2}) at the negative and positive electrode/SSE interfaces are described by Butler-Volmer equation, i.e.,

$$j_{\text{loc,ne}} = \gamma \underbrace{F k_{\text{ne}} \left(c_{\text{Li}^+} \right)^{\alpha_a} \left(\bar{c}_{\text{Li,ne}} \right)^{(1-\alpha_a)}}_{j_{0,\text{ne}}} \left(\exp\left(\frac{\alpha_a F \eta_{\text{ne}}}{RT} \right) - \exp\left(-\frac{\alpha_c F \eta_{\text{ne}}}{RT} \right) \right) \qquad \text{(Equation S6A)}$$

and

$$j_{\text{loc,pe}} = \gamma \underbrace{F k_{\text{pe}} \left(c_{\text{Li,pe}}^{\max} - c_{\text{Li,pe}} \right)^{\alpha_c} \left(c_{\text{Li,pe}} \right)^{(1-\alpha_c)} \left(c_{\text{Li}^+} \right)^{\alpha_c}}_{j_{0,\text{pe}}} \left(\exp\left(\frac{\alpha_a F \eta_{\text{pe}}}{RT} \right) - \exp\left(-\frac{\alpha_c F \eta_{\text{pe}}}{RT} \right) \right) \text{(Equation S6B)}$$

where k_{ne} (m s^{-1}) and k_{pe} (m$^{2.5}$ mol$^{-0.5}$ s^{-1}) are the reaction rate constants for negative and positive reactions, respectively, γ the contact area ratio, $\eta_{\text{ne/pe}}$ (V) the overpotential at the electrode/SSE interfaces, expressed by

$$\eta_{\text{ne}} = \phi_e - \phi_{\text{ion}} \qquad \text{(Equation S7A)}$$

and

$$\eta_{\text{pe}} = \phi_e - \phi_{\text{ion}} - U_{\text{eq,pe}} \qquad \text{(Equation S7B)}$$

where $U_{\text{eq,pe}}$ (V) is the equilibrium potential for the $LiCoO_2$ cathode, which can be fitted to a

polynomial function, written as[3]

$$U_{\mathrm{eq,pe}}\left(soc\right)=\frac{-4.656+88.669soc^{2}-401.119soc^{4}+342.909soc^{6}-462.471soc^{8}+433.434soc^{10}}{-1+18.933soc^{2}-79.532soc^{4}+37.311soc^{6}-73.083soc^{8}+95.96soc^{10}}$$

(Equation S8)

where *soc*, i.e., state of charge, is defined as

$$soc=\frac{c_{\mathrm{Li,pe}}}{c_{\mathrm{Li,pe}}^{\max}} \quad \text{(Equation S9)}$$

Table S1 Source terms of conservation equations

Source terms	Unit
$S_{\mathrm{Li,pe}}=\begin{cases}0 & \text{other zones}\\ -\dfrac{j_{\mathrm{loc}}A_{\mathrm{cell}}}{\gamma F V_{\mathrm{cell}}} & \text{boundary mesh of interface 3 for positive electrode}\end{cases}$	mol m^{-3} s^{-1}
$S_{\mathrm{Li^{+}}}=\begin{cases}0 & \text{other zones}\\ \left(1-\dfrac{D_{+}}{D_{+}+D_{-}}\right)\dfrac{j_{\mathrm{loc}}A_{\mathrm{cell}}}{\gamma F V_{\mathrm{cell}}} & \text{boundary mesh of interface 2,3 for SSE}\end{cases}$	mol m^{-3} s^{-1}
$S_{\mathrm{Li^{+}}}^{\mathrm{ionize}}=\begin{cases}0 & \text{other zones}\\ k_{r}\left(\delta^{2}{c_{0}}^{2}-{c_{\mathrm{Li^{+}}}}^{2}\right) & \text{SSE}\end{cases}$	mol m^{-3} s^{-1}
$S_{\mathrm{e}}=\begin{cases}0 & \text{other zones}\\ -\dfrac{j_{\mathrm{loc}}A_{\mathrm{cell}}}{V_{\mathrm{cell}}} & \text{boundary mesh of interface 2,3 for electrodes}\end{cases}$	A m^{-3}
$S_{\mathrm{ion}}=\begin{cases}0 & \text{other zones}\\ \dfrac{j_{\mathrm{loc}}A_{\mathrm{cell}}}{V_{\mathrm{cell}}} & \text{boundary mesh of interface 2,3 for SSE}\end{cases}$	A m^{-3}

Initial and boundary conditions

Initially, the lithium/lithium-ion concentration and the electronic/ionic potential boundary conditions are set as follows,

$$c_{\mathrm{Li}^+} = \delta c_0, c_{\mathrm{Li,pe}} = c_{\mathrm{Li,pe}}^{\mathrm{min}}$$
$$\phi_{\mathrm{ion,SSE}} = 0, \phi_{\mathrm{e,Cu}} = \phi_{\mathrm{e,ne}} = 0, \phi_{\mathrm{e,pe}} = \phi_{\mathrm{e,Al}} = U_{\mathrm{eq,pe}}(soc = 0.5) \quad \text{(Equation S10)}$$

The cut-off voltage, once reached which the discharge will be stopped, is set to be 3.5 or 3.0 V. Ground boundary and galvanostatic condition are adopted for the terminal boundary conditions of negative and positive current collectors, respectively, which are defined as,

$$\phi_e |_{\mathrm{terminal,ne}} = 0 \quad \text{(Equation S11A)}$$

and

$$-\sigma_{\mathrm{e}} \nabla \phi_{\mathrm{e}} |_{\mathrm{terminal,pe}} = I_{\mathrm{app}} \quad \text{(Equation S11B)}$$

Performance indicators

To assess the performance of the battery with 3D pillar electrode, three performance indicators, including area-specific energy density, power density and capacity, are defined as follows:

Energy density (mWh cm^{-2})

$$E = \frac{\int_0^t I_{\mathrm{app}} V \mathrm{d}t}{3600} \times 0.1 \quad \text{(Equation S12A)}$$

Power density (mW cm^{-2})

$$P = \frac{\int_0^t I_{\mathrm{app}} V \mathrm{d}t}{t} \times 0.1 \quad \text{(Equation S12B)}$$

Capacity (mAh cm^{-2})

$$Cap = \frac{\int_0^t I_{\mathrm{app}} \mathrm{d}t}{3600} \times 0.1 \quad \text{(Equation S12C)}$$

Numerical procedures

To simulate specific physical processes, the model governing equations are solved with the specific boundary conditions, geometric dimensions, operating conditions, and physicochemical properties listed in Table S2. An open-source software based on finite volume method,

OpenFOAM, with SIMPLE algorithm is used to solve these governing equations. The convergence criterions of all the physical quantities are set to 1×10^{-6}. The anode, SSE and cathode are divided into 5, 10 and 10 layers of grid along the thickness direction, respectively. For the 3D electrode, the grid length is set to 1 μm along the height direction, and thus the total grid numbers change with the height of pillars. The grid independence test has been conducted successfully with the discharge time step of 1 s.

Table S2 Geometric dimensions, operating conditions and physicochemical properties

Parameters	Symbol	Value	Unit
Thickness of negative electrode/SSE/positive electrode[1]	$L_{ne}/L_{SSE}/L_{pe,}$	10/3.62/8.08	μm
Conductivity of SSE/positive electrode[4,5]	σ_{SSE}/σ_{pe}	5.9×10^{-4}/0.01	S m^{-1}
Temperature[1]	T	293	K
Recombination rate[1]	k_r	8×10^{-7}	m^3 mol^{-1} s^{-1}
Negative reaction rate constant[1]	k_{ne}	1.09×10^{-9}	m s^{-1}
Positive reaction rate constant[1]	k_{pe}	1.53×10^{-11}	$m^{2.5}$ $mol^{-0.5}$ s^{-1}
Transfer coefficient[1]	α_a/α_c	0.5/0.5	
Diffusion coefficient of cation/anion[1]	D_+/D-	$1.73\times10^{-16}/5.69\times10^{-16}$	m^2 s^{-1}
Diffusion coefficient of lithium[1]	$D_{Li,pe}$	1.04×10^{-13} ($soc\leq0.7$)	m^2 s^{-1}
		Interpolate ($0.7<soc<0.92$)	m^2 s^{-1}
		5.38×10^{-15} ($soc\geq0.92$)	m^2 s^{-1}
Maximum/minimum lithium concentration[1]	$c_{Li,pe}^{max}/c_{Li,pe}^{min}$	32200/16100	mol m^{-3}
Total amount of lithium in SSE[1]	c_0	61100	mol m^{-3}

Mole fraction of mobile lithium-ion[1]	δ	0.64	
Discharge current	I_{app}	3.487 (1 C)/10	A m^{-2}
Pillar height	h	50/100/150/200	μm

Note S3 Model validation

To validate this model, the discharge curves of partial cell and whole cell are compared with the experimental data[2] (Figure S3a), of which the whole cell data considers the non-uniform current distribution caused by tabs. The simulation results agree well with the experimental data over the whole discharge process. The deviation between the numerical results and the experimental data could be attributed to the assumptions of uniform distribution of lithium-ion in the anode and the neglect of side reactions. Besides, it should be mentioned that it is because of the negligible inhomogeneity of small size battery that the discharge curves of the partial cell are nearly coincided with that of the whole cell.

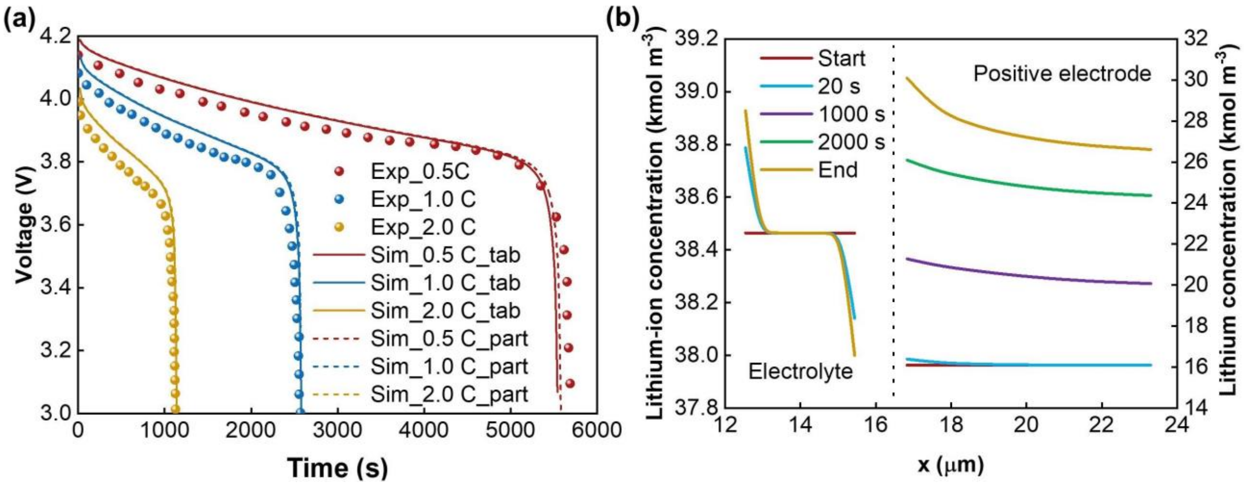

Figure S3. Model validation. (a) Comparisons of the simulated discharge curves with the experiment results[1]; (b) Concentration profiles in the solid-state electrolyte and positive electrode of the partial cell along the thickness direction at 1 C.

Figure S3b shows the development of concentration profiles of the mobile lithium-ion in the

SSE (on the left) and the intercalated lithium-ion in the cathode along the thickness direction of the partial cell at 1 C discharge, which exhibit the same trend as the published works.[1,6] It is seen that the distribution of lithium-ion concentration in the SSE almost remains steady in the process of discharge, except at the beginning. The mobile lithium-ion concentration gradient is relatively high near the SSE/electrode interfaces but almost zero in the inner region. The lithium concentration in the cathode increases with time due to the continuous intercalation at the SSE/positive electrode interface and inward diffusion of lithium-ion. Since the diffusion of lithium is driven by the concentration gradient, the lithium concentration in the cathode is small at the place away from the SSE/positive electrode interface, which could result in a low utilization rate of the active material when the electrode thickness or discharge rate is large.

Figure S4 illustrates the distribution of electron potential within current collector. It is observed that the place with the highest potential in the anode is on the terminal of the negative tab, and the place with the lowest potential in the cathode is located at the terminal of the positive tab. Besides, the potential gradient is more significant at the place near tab terminal, which indicates a higher local electric current near and within the tab.

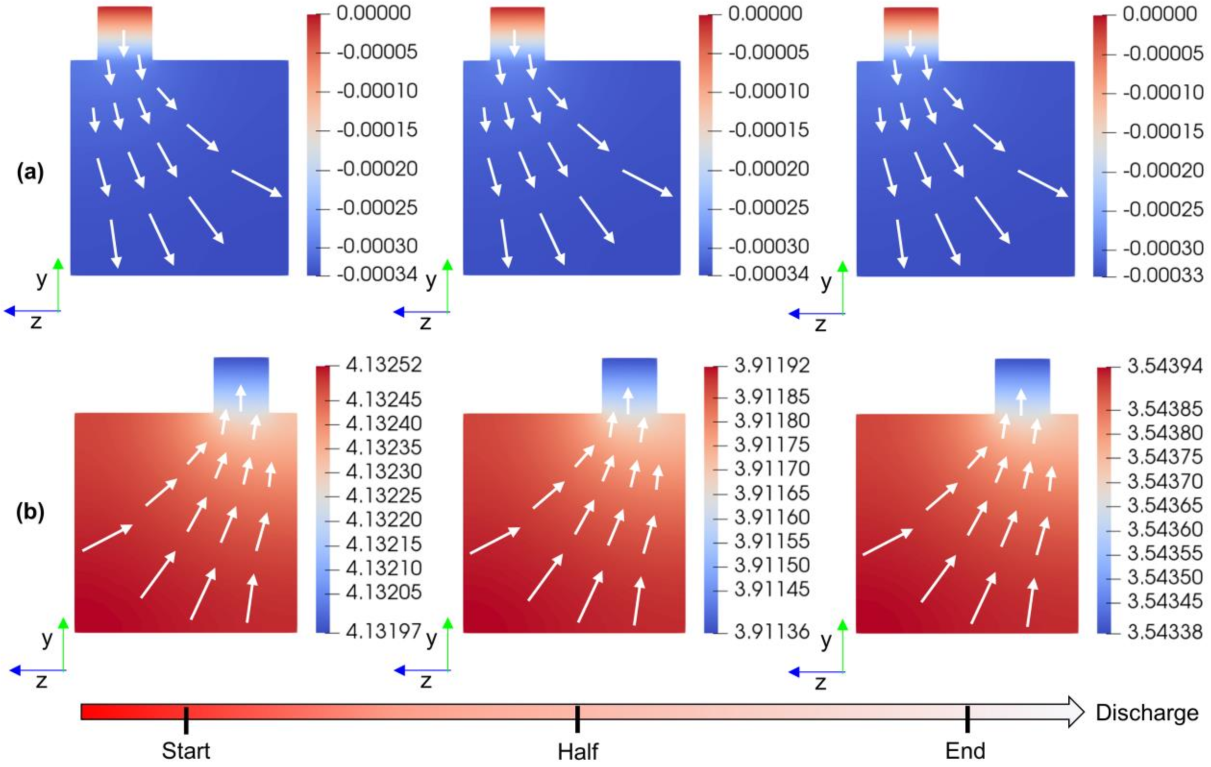

Figure S4. Local distribution of electric potential within current collector at 1 C. (a) Negative side; (b) Positive side.

The local distribution of overpotential at the electrode/SSE interfaces is shown in Figure S5. It is found that the overpotential distribution is dominantly influenced by the potential distribution of positive electrode since the electrical conductivity is larger in the lithium metal anode. In other words, the potential gradient is lower in the anode. In addition, while the magnitude of the anode interface overpotential almost remains constant, the cathode interface overpotential increases with the depth of discharge. The interface overpotential of the cathode has a larger contribution than that of the anode to the total overpotential. The distribution of overpotential has a significant change with the progress of discharge. At the beginning and middle stage of discharge, the region located in the upper and right part of the cell has a higher overpotential value. However, at the end stage of discharge, the upper and right part of the cell turns out to have the lowest overpotential value of the whole cell.

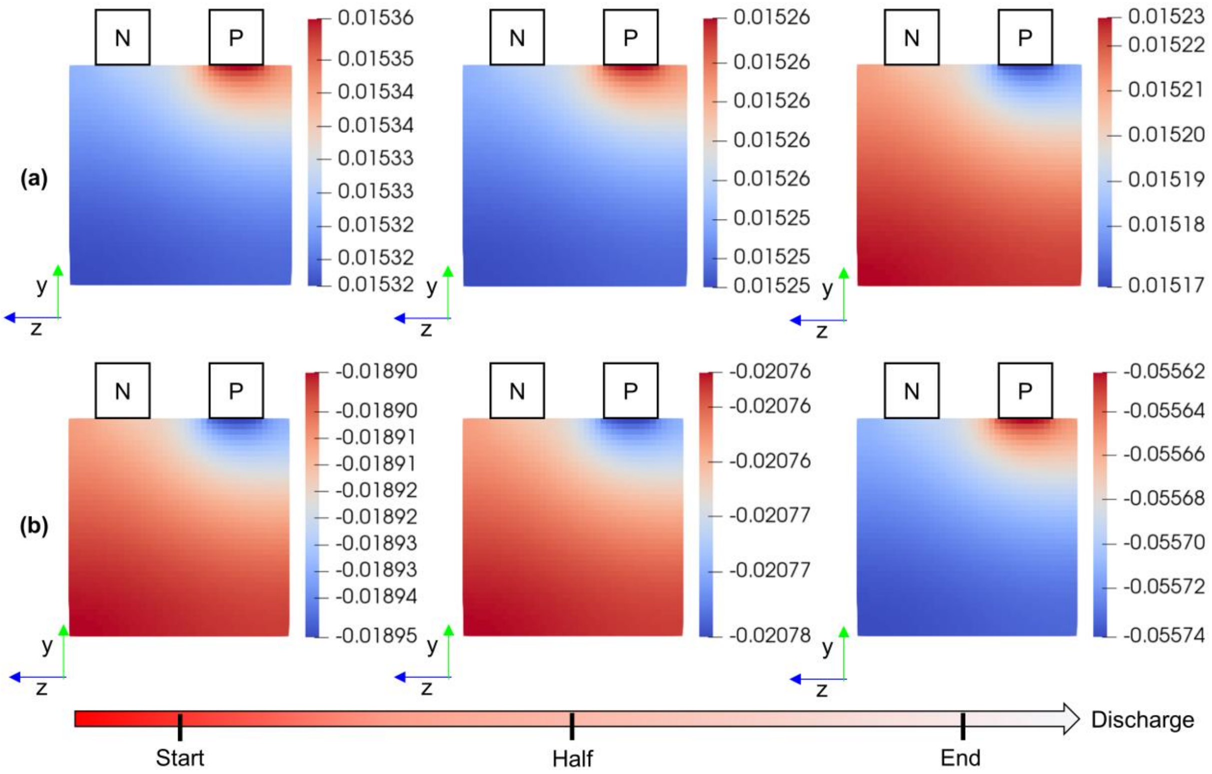

Figure S5. Local distribution of the electrode/solid-state electrolyte interface overpotential at 1 C. (a) Negative side; (b) Positive side.

Supplemental References

[1] L.H.J. Raijmakers, D.L. Danilov, R.A. Eichel, P.H.L. Notten, *Electrochim. Acta* 2020, 330, 135147.

[2] M. Xu, Z. Zhang, X. Wang, L. Jia, L. Yang, *J. Power Sources* 2014, 256, 233-243.

[3] S. Tang, Z. Wang, H. Guo, J. Wang, X. Li, G. Yan, *Solid State Ion.* 2019, 343,115083.

[4] Y. Zheng, Y. Yao, J. Ou, M. Li, D. Luo, H. Dou, Z. Li, K. Amine, A. Yu, Z. Chen, *Chem. Soc. Rev.* 2020, 49, 8790.

[5] P. Priimägi, D. Brandell, S. Srivastav, A. Aabloo, H. Kasemägi, V. Zadin, *Electrochim. Acta* 2019, 209, 138-148.

[6] N. Kazemi, D.L. Danilov, L. Haverkate, N.J. Dudney, S. Unnikrishnan, P.H.L. Notten, *Solid State Ion.* 2019, 334,111.
